# Efficacy of radial extracorporeal shock wave therapy for chronic prostatitis/chronic pelvic pain syndrome: A protocol for systematic review

**DOI:** 10.1097/MD.0000000000022981

**Published:** 2020-10-30

**Authors:** Guangsen Li, Degui Chang, Di’ang Chen, Peihai Zhang, Yaodong You, Xiaopeng Huang, Jian Cai, Xuesong Yang

**Affiliations:** Hospital of Chengdu University of Traditional Chinese Medicine, Chengdu, Sichuan Province, China.

**Keywords:** chronic prostatitis/chronic pelvic pain syndrome, protocol, radial extracorporeal shock wave therapy

## Abstract

**Background:**

Prostatitis is a common urogenital system disease in men which affects 5% to 9% of adult men worldwide and accounts for approximately 8% of visits to urologists. In the past years, its pathogenesis is complicated and the classification of it is not clear, so the effect of treatment measures is not significant. Recently, the treatment of chronic prostatitis/chronic pelvic pain syndrome (CP/CPPS) includes nonsteroidal anti-inflammatory drugs, phytotherapy, hormonal therapy, alpha-blockers, anti-anxiolytic, and acupuncture, which provide more choice for the urologist. But there still are some limitations. scholars. Many studies suggest radial extracorporeal shock wave therapy may be the better option in the treatment of CP/CPPS. However, the efficacy and safety of it still lack solid evidence.

**Methods and analysis:**

The electronic databases of MEDLINE, PubMed, Web of Science, EMBASE, Cochrane Library, Clinicaltrials.org, China National Knowledge Infrastructure Database, Wan fang Database, China Biology Medicine Database, VIP Science Technology Periodical Database, Chinese Clinical Trial Registry will be retrieved. All the randomized controlled trials of radial extracorporeal shock wave therapy (rESWT) for patients with CP/CPPS will be included. We will evaluate the outcomes including National Institutes of Health Chronic Prostatitis Symptom Index, visual analog scale, international prostate symptom score, international index of erectile function-5, and conduct this study strictly according to the Cochrane Handbook for Systematic Reviews of Interventions.

**Results:**

The current study is a protocol for systematic review and meta-analysis without results, and data analysis will be carried out after the protocol. We will share our findings on October 31st of 2021.

**Conclusion:**

rESWT as a noninvasive treatment with no pain, which will be accepted more easily. Although some studies have suggested that rESWT can relieve the symptoms of patients, the efficacy and safety of it still lack solid evidence. To address this limitation scientifically and systematically, this study will inspect the efficacy and safety of the rESWT treatment in patients with CP/CPPS by integrating various studies.

**Ethics and dissemination:**

Formal ethical approval is not required in this protocol. We will collect and analyze data based on published studies, and since there are no patients involved in this study, individual privacy will not be under concerns. The results of this review will be disseminated to peer-reviewed journals or submit to related conferences.

**Protocol registration number:**

INPLASY202090076

## 1 Introduction

Prostatitis is a common urogenital system disease in men. It has been the 3rd most commonly found urinary tract disease in men after benign prostate hyperplasia and prostate cancer.^[1]^ Overall, the prevalence of prostatitis ranges from 5% to 9%,^[2]^ and prostatitis accounts for approximately 8% of visits to urologists in the USA.^[3]^ In the past years, its pathogenesis is complicated and the classification of it is not clear, so the effect of treatment measures is not significant.

In the late 1990s, to solve this problem, the National Institute of Health (NIH)^[4]^ divides prostatitis into Type I-IV, and type III prostatitis which also called Chronic prostatitis/chronic pelvic pain syndrome (CP/CPPS) is divided into inflammatory chronic pelvic pain syndrome (IIIa) and non-inflammatory chronic pelvic pain syndrome (IIIb) and the National Institute of Health-Chronic Prostatitis Symptom Index (NIH-CPSI) score^[5]^ is a validated measure commonly used to measure CP/CPPS symptoms. CP/CPPS exists in more than 90% to 95% of patients with prostatitis.^[6]^ CP/CPPS is used to define and include many different symptomatological patterns, and its understanding is still enigmatic for many physicians and patients. The common point of view agrees with the syndrome of CP/CPPS includes pelvic pain,^[7]^ genital pain,^[8]^ obstructive voiding difficulties,^[9]^ ejaculatory pain, and erectile dysfunction.^[10]^ It is CP/CPPS that provides the most frustration for patients and practitioners, and it even leads to divorce.^[11]^

In 2009, the urinary psychosocial, organ specific, infection, neurologic, and tenderness of skeletal muscle classification system was proposed for patients who have been diagnosed with CP/CPPS.^[12]^ It consists of 6 points that are designed to classify patients with an established diagnosis of CP/CPPS into one or more of the 6 clinically determining areas that can rationally guide therapy. The identifiable areas are as follows: urinary, psychosocial, organ-specific, infection, neurologic/systemic, and tenderness (muscle). This could give help for the treatment of CP/CPPS.

Until recently, the treatment of CP/CPPS include NSAIDs,^[13]^ phytotherapy,^[14]^ hormonal therapy,^[15]^ alpha-blockers,^[16]^ anti-anxiolytic,^[17]^ and acupuncture,^[18]^ which provide more choice for the urologist. But these therapies have various side effects, physiotherapy^[5]^ as a new noninvasive treatment attaches more and more attentions of scholars. Some studies^[19,20]^ suggest radial extracorporeal shock wave therapy (rESWT) may be the better option in the treatment of CP/CPPS. However, the efficacy and safety of it have not yet been fully investigated, we still lack adequate evidence to confirm these.

## 2 Objectives

The purpose of this study is to further evaluate the efficacy and safety of radial extracorporeal shock wave therapy in the treatment of CP/CPPS. The results will provide urologists and andrologists with clinical decisions.

## 3 Methods

The protocol was registered on the International Platform of Registered Systematic Review and Meta-analysis Protocols (registration number: INPLASY202090076) which could be available on https://inplasy.com. The content refers to the statement of the Preferred Reporting Items for Systematic Review and Meta-Analysis Protocols checklist.^[21]^

### 3.1 Eligibility criteria

The inclusion and exclusion criteria are as follows.

#### 3.1.1 Types of Studies

All the randomized controlled trials of rESWT for patients with CP/CPPS will be included without publication status restriction or writing language letters to editors, review articles, case reports, conference abstracts, cross-sectional studies, and all observational studies will be excluded.

#### 3.1.2 Participants

Inclusion criteria:

- NIH-CPSI >19, and a pain score of NIH-CPSI >3.
- Pain in penis, testicles, perineum, or lumbosacral region.
- Voiding symptoms, such as dysuria, frequency, and sense of incomplete urination.
- The minimum duration of these symptoms for inclusion in the study was 3 months.

Exclusion criteria:

- Patients with a past medical history for urinary tract infections.
- Patients with urethral malformation, stricture, urinary calculi, and cystitis.
- Patients with benign prostate hyperplasia, prostate cancer, epididymitis, urinary tract tuberculosis, and spermatic cord disease.
- Patients with mental illness.

#### 3.1.3 Types of interventions and controls

Experimental interventions:

The patients in the treatment group received rESWT.

Control interventions:

The control group could gain a nonsteroidal anti-inflammatory drugs, phytotherapy, hormonal therapy, alpha-blockers, anti-anxiolytics, acupuncture, or guideline-recommended conventional treatment.

#### 3.1.4 Types of outcome measures

Primary outcome:

(1) NIH-CPSI.

Secondary outcomes:

(1) Visual analog scale (0–10)
(2) Micturition conditions were examined using the international prostate symptom score (0–35)
(3) The international index of erectile function-5 (0–25) was applied for evaluating erectile dysfunction.

### 3.2 Search strategy

#### 3.2.1 Data sources

The electronic databases of MEDLINE, PubMed, Web of Science, EMBASE, Cochrane Library, Clinicaltrials.org, China National Knowledge Infrastructure Database, Wan fang Database, China Biology Medicine Database, VIP Science Technology Periodical Database, Chinese Clinical Trial Registry will be retrieved. They will be searched until June 2021 to recognize related studies. The search strategy that will be run in the PubMed and adjusted to fit the other database when necessary is presented in Table 1.

**Table 1:**
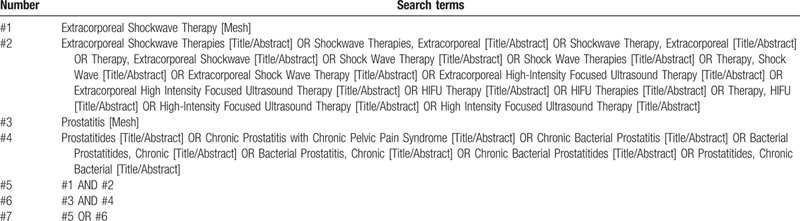
This table presents the initial draft of the search strategy with PubMed as an example.

#### 3.2.2 Other sources of search

Grey literature will be retrieved through Open Grey. Besides, we will also scan the reference lists of manual review articles for any possible titles matching the inclusion criteria.

### 3.3 Data extraction, quality, and validation

#### 3.3.1 Study inclusion

**Figure 1:**
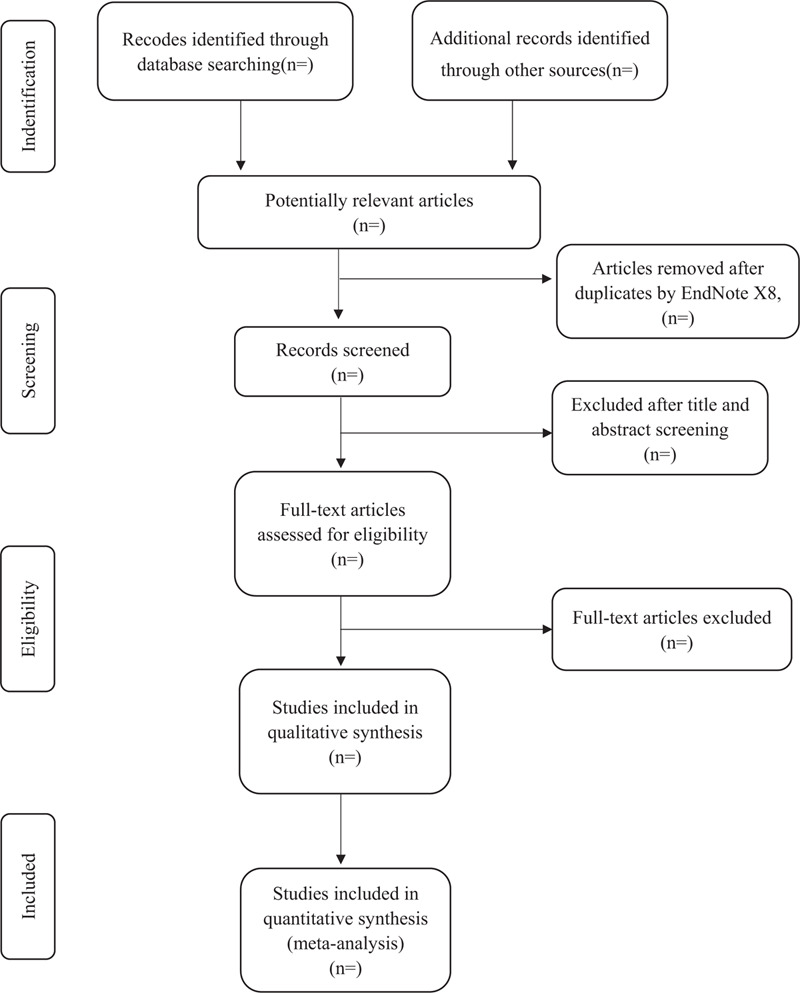
Study selection flow chart.

According to pre-defined eligibility criteria, importing the literature retrieved to the Endnote X8 and eliminate the duplicate data. The software will be used to filter duplicate documents first, and then the studies which don’t meet the inclusion criteria will be removed. If the studies appear to meet the inclusion criteria or there is any uncertainty based on the information provided in the title and abstract, full texts will be obtained for further assessment. Further detailed screening and data extraction of the documents will be performed simultaneously by 2 professionally trained reviewers. When necessary, the original study author will be contacted for judgment. Disagreements will be resolved by discussion or taking the expert (GSL) for arbitration. The number and reasons for excluding trials will be recorded in detail. A flow diagram of the study selection is shown in Figure 1.

#### 3.3.2 Data extraction and management

The two reviewers will independently read and extract the data from the study. Before the formal process of data extraction, the review group will discuss and a unified data extraction form (an excel spreadsheet) will be produced. The content data will include the following information: title, abstract, first author and corresponding author, the country, the publishing year, publications, participants, demographic characteristics (age, family situation, regional, ethnic, and national), the number of participants, diagnostic criteria, types, intervention, observation index (NIH-CPSI, visual analog scale, international prostate symptom score, international index of erectile function-5), the results of the study, the incidence of adverse events and type. All disagreement between the 2 reviewers will be decided by consensus or with the participation of a third reviewer. Besides, we will also contact the author via email to request any missing data or clarification. If we cannot obtain the missing data, we will report it in the risk assessment of bias and consider its impact on the analysis of the data.

### 3.4 Risk of bias assessment

We will evaluate selection bias, detection bias, attrition bias, performance bias, and other bias based on the Cochrane Collaboration Network Risk Assessment Tool. The tool assesses the risk of bias mainly in the following 7 aspects: random sequence generation, allocation concealment, the blinding method for patients, researchers and outcomes assessors, incomplete result data, and selective reports. As recommended by the Cochrane manual, the risk of bias in each of these areas will be assessed as low or high depending on whether the criteria were met or not met, and the lack of information will be recorded as unclear. The risk of bias will be checked by 2 review authors. Discrepancies between review authors on the risk of bias will be resolved through discussion with a third review author.

### 3.5 Quantitative data synthesis and statistical methods

#### 3.5.1 Data analysis and synthesis

The RevMan5.3 software will be used to conduct the meta-analysis (If feasible). Descriptive analysis or systematic narrative synthesis will be performed to summarize and explain the characteristics and findings of the included studies and provide the information in the texts and tables. For dichotomous data (eg, effective and ineffective), we will calculate risk ratio and 95% confidence intervals. For continuous data, which will be pooled as mean difference.

#### 3.5.2 Investigation of heterogeneity

The *Q* statistic and *I*^*2*^ statistic of Cochran will be used for testing heterogeneity. If *P* ≤ .10 or *I*^*2*^ ≥ 50%, heterogeneity will be considered significant. At this point, a fixed effects model (Mantel–Haenzel method for risk ratio and Inverse Variance for mean difference) will be used for *I*^2^ < 50%. A random effects model (D-I method) will be used when the heterogeneity is still significant after sensitivity analysis and subgroup analysis.

#### 3.5.3 Subgroup analysis

If necessary, we will identify the source of heterogeneity through subgroup analysis and manage the heterogeneity:

(1) The duration and severity of CP/CPPS.
(2) whether with other complications.
(3) demographic characteristics of the patients: age, marital and family status, region, race.
(4) follow-up time.

#### 3.5.4 Sensitivity analysis

Sensitivity analysis will be used to test the reliability and stability of the meta-analysis results, and to assess the source of heterogeneity. We will compare the results before and after by excluding trials with a high risk of bias or eliminating each study individually one study each time and then pooling the remaining studies.

#### 3.5.5 Grading the quality of evidence

The grading of recommendations assessment, development, and evaluation tool^[22]^ will be applied to judge the quality of evidence in the systematic review. It consists of risk of bias, consistency, directness, precision, and publication bias. Two independent reviewers will assess these studies. In most cases, disagreements were resolved by discussion between the 2 reviewers. If disagreement remained after discussion, the third reviewer will be consulted before taking the final decision on the disagreements.

#### 3.5.6 Publication bias

Published bias will be measured by the funnel plot. If the result is indistinct, the Begg test and Egger test will be used (by STATA software 11.0).

#### 3.5.7 Reporting of the review

The quality of the manuscript will be standardized by each item of the AMSTAR-2 tool. And the results will be reported following the Preferred Reporting Items for Systematic Reviews and Meta-Analysis statement.^[23]^

## 4 Discussion

rESWT for CP/CPPS is a noninvasive treatment with no pain. Resulting from this, we believe that rESWT will be accepted by most patients. Hence, the efficacy and safety of rESWT in urgent need to be proved. These prior results have suggested that rESWT can improve the NIH-CPSI score. But the sample of these still is small and lacking safety of them. In order to address this limitation scientifically and systematically, this study will inspect the efficacy and safety of the rESWT treatment in patients with CP/CPPS by integrating various studies. Despite that, this review still exists some limitations. Because of the different symptoms of CP/CPPS, the assessment of rESWT may exist deviation.

## Author contributions

**Conceptualization:** Guangsen Li, Xuesong Yang.

**Data curation:** Degui Chang, Di’ang Chen.

**Formal analysis**: Yaodong You, Xiaopeng Huang.

**Methodology:** Peihai Zhang, Yaodong You.

**Project administration:** Yaodong You, Xiaopeng Huang.

**Software:** Guangsen Li, Degui Chang, Jian Cai.

**Supervision:** Di’ang Chen, Xiaopeng Huang.

**Validation:** Peihai Zhang, Yaodong You.

**Writing – original draft:** Guangsen Li, Jian Cai.

**Writing – review & editing:** Guangsen Li, Xuesong Yang.

## References

[1] Collins MM Stafford RS O’leary MP How common is prostatitis? A national survey of physician visits. J Urol 1998;159:1224–8. 9507840

[2] Krieger JN Lee SW Jeon J Epidemiology of prostatitis. Int J Antimicrobial Agents 2008;31: Suppl 1: S85–90. 10.1016/j.ijantimicag.2007.08.028 PMC2292121 18164907

[3] Zhang R Sutcliffe S Giovannucci E Lifestyle and risk of chronic prostatitis/chronic pelvic pain syndrome in a cohort of United States male health professionals. J Urol 2015;194:1295–300. 26070893 10.1016/j.juro.2015.05.100 PMC4666310

[4] Krieger JN Nyberg L Nickel JR NIH consensus definition and classification of prostatitis. JAMA 1999;282:236–7. 10422990 10.1001/jama.282.3.236

[5] Rees J Abrahams M Doble A Diagnosis and treatment of chronic bacterial prostatitis and chronic prostatitis/chronic pelvic pain syndrome: a consensus guideline. BJU Int 2015;116:509–25. 25711488 10.1111/bju.13101 PMC5008168

[6] Khan FU Ihsan AU Khan HU Comprehensive overview of prostatitis. Biomed Pharmacother 2017;94:1064–76. 28813783 10.1016/j.biopha.2017.08.016

[7] Bharucha AE Lee TH Anorectal and pelvic pain. Mayo Clin Proc 2016;91:1471–86. 27712641 10.1016/j.mayocp.2016.08.011 PMC5123821

[8] Biasi G Di Sabatino V Ghizzani A Chronic pelvic pain: comorbidity between chronic musculoskeletal pain and vulvodynia. Reumatismo 2014;66:87–91. 24938200 10.4081/reumatismo.2014.768

[9] Hu M Wazir J Ullah R Phytotherapy and physical therapy in the management of chronic prostatitis-chronic pelvic pain syndrome. Int Urol Nephrol 2019;51:1081–8. 31054003 10.1007/s11255-019-02161-x

[10] Tran CN Shoskes DA Sexual dysfunction in chronic prostatitis/chronic pelvic pain syndrome. World J Urol 2013;31:741–6. 23579441 10.1007/s00345-013-1076-5

[11] Mehik A Hellström P Sarpola A Fears, sexual disturbances and personality features in men with prostatitis: a population-based cross-sectional study in Finland. BJU Int 2001;88:35–8. 10.1046/j.1464-410x.2001.02259.x 11446842

[12] Shoskes DA Nickel JC Dolinga R Clinical phenotyping of patients with chronic prostatitis/chronic pelvic pain syndrome and correlation with symptom severity. Urology 2009;73:538–42. 19118880 10.1016/j.urology.2008.09.074

[13] Yellepeddi VK Radhakrishnan J Radhakrishnan R Penetration and pharmacokinetics of non-steroidal anti-inflammatory drugs in rat prostate tissue. Prostate 2018;78:80–5. 29105796 10.1002/pros.23447

[14] Gupta SC Patchva S Aggarwal BB Therapeutic roles of curcumin: lessons learned from clinical trials. AAPS J 2013;15:195–218. 23143785 10.1208/s12248-012-9432-8 PMC3535097

[15] Nickel JC Downey J Pontari MA A randomized placebo-controlled multicentre study to evaluate the safety and efficacy of finasteride for male chronic pelvic pain syndrome (category IIIA chronic nonbacterial prostatitis). BJU Int 2004;93:991–5. 15142149 10.1111/j.1464-410X.2003.04766.x

[16] Nickel JC Narayan P Mckay J Treatment of chronic prostatitis/chronic pelvic pain syndrome with tamsulosin: a randomized double blind trial. J Urol 2004;171:1594–7. 15017228 10.1097/01.ju.0000117811.40279.19

[17] Shulyak A Gorpynchenko I Drannik G The effectiveness of the combination of rectal electrostimulation and an antidepressant in the treatment of chronic abacterial prostatitis. Central Eur J Urol 2019;72:66–70. 10.5173/ceju.2018.1719 PMC6469008 31011444

[18] Qin Z Zang Z Zhou K Acupuncture for chronic prostatitis/chronic pelvic pain syndrome: a randomized, sham acupuncture controlled trial. J Urol 2018;200:815–22. 29733836 10.1016/j.juro.2018.05.001

[19] Salama AB Abouelnaga WA Effect of radial shock wave on chronic pelvic pain syndrome/chronic prostatitis. J Physical Therapy Sci 2018;30:1145–9. 10.1589/jpts.30.1145 PMC6127492 30214114

[20] Zhang Z-X Zhang D Yu X-T Efficacy of radial extracorporeal shock wave therapy for chronic pelvic pain syndrome: a nonrandomized controlled trial. Am J Men's Health 2018;13:1557988318814663. 30486723 10.1177/1557988318814663 PMC6775558

[21] Moher D Shamseer L Clarke M Preferred reporting items for systematic review and meta-analysis protocols (PRISMA-P) 2015 statement. Syst Rev 2015;4:1. 25554246 10.1186/2046-4053-4-1 PMC4320440

[22] Guyatt GH Oxman AD Vist GE GRADE: an emerging consensus on rating quality of evidence and strength of recommendations. BMJ (Clinical Research Ed) 2008;336:924–6. 10.1136/bmj.39489.470347.AD PMC2335261 18436948

[23] Moher D Liberati A Tetzlaff J Preferred reporting items for systematic reviews and meta-analyses: the PRISMA statement. PLoS Med 2009;6:e1000097. 19621072 10.1371/journal.pmed.1000097 PMC2707599

